# Atomic-Scale Mechanisms of Ultrasonic-Assisted Ultra-Precision Cutting of W-Mo70 Alloy: Experimental Benchmarking and Molecular Dynamics Simulation

**DOI:** 10.3390/mi17091076

**Published:** 2026-09-11

**Authors:** Yonglin Min, Zhanjie Li, Gang Jin

**Affiliations:** College of Mechanical Engineering, Tianjin University of Technology and Education, Tianjin 300222, China

**Keywords:** W-Mo70 alloy, ultra-precision cutting, ultrasonic elliptical vibration cutting, molecular dynamics, vibration-induced transient loading–unloading response, dislocation evolution, subsurface deformation

## Abstract

W-Mo70 alloys feature high hardness, a high melting point and poor machinability, resulting in large cutting loads and complex subsurface plastic deformation during ultra-precision machining. To reveal the atomic-scale mechanism by which ultrasonic elliptical vibration regulates material removal and defect evolution, three-dimensional molecular dynamics (MD) models of conventional cutting (CC) and ultrasonic elliptical vibration cutting (UEVC) were established with previously published ultra-precision turning experiments as the macroscopic benchmark. The material removal behavior, cutting force response, interfacial loading characteristics and dislocation evolution law were systematically analyzed. The results show that UEVC exhibits a reduction trend in the cycle-averaged main cutting force compared with CC, which is qualitatively consistent with the experimental trend. However, the reduction magnitude should not be directly compared with experimental results because of the significant differences in machining scale, cutting velocity, strain rate, and tool geometry between MD simulations and experiments. The average normal force remains nearly unchanged but exhibits a significant vibration-induced transient loading–unloading response. Under UEVC, dislocation evolution transforms from continuous accumulation under CC to transient activation at the high-load stage and defect reorganization during the unloading stage. This study reveals the atomic-scale mechanism of UEVC characterized by a vibration-induced transient loading–unloading response, interfacial unloading, defect reorganization and phase-dependent localized material removal, providing atomic-level theoretical support for subsurface defect regulation in ultra-precision cutting of difficult-to-machine W-Mo alloys.

## 1. Introduction

Tungsten and tungsten-based alloys are widely used in nuclear engineering, aerospace, high-energy physics, precision optical molds, and specialized functional devices because of their high melting point, high density, excellent high-temperature strength, wear resistance, irradiation resistance, and dimensional stability [1]. However, tungsten generally exhibits high hardness and limited ductility at room temperature, and its plastic deformation capability is strongly governed by dislocation motion. The relatively low mobility of dislocations tends to result in high cutting resistance, localized stress concentration, and brittle damage during machining [2]. These challenges become more pronounced under ultra-precision cutting conditions, where the characteristic scale of the tool edge approaches that of the local material deformation zone. Consequently, material removal is jointly affected by tool geometry, depth of cut, crystal structure, and dislocation activity, making the control of surface and subsurface integrity particularly difficult. Previous nanoscale cutting studies have shown that the plastic deformation of tungsten can be accommodated by both dislocation slip and local structural disordering, among which the nucleation and propagation of 1/2⟨111⟩ dislocations constitute an important component of its atomic-scale plastic response [3].

To improve the machinability of difficult-to-cut materials, vibration-assisted machining superimposes high-frequency and small-amplitude motion on the conventional cutting movement, thereby actively modulating the transient tool–workpiece interfacial contact state. Shamoto and Moriwaki first proposed the elliptical vibration cutting method and demonstrated that a two-dimensional vibration trajectory can significantly alter chip formation and cutting force response [4]. Subsequently, Brehl and Dow systematically reviewed the kinematics, tool–workpiece contact behavior, and machining performance of vibration-assisted machining [5], while Xu and Zhang and Yang et al. further summarized ultrasonic vibration-assisted machining from the perspectives of vibration devices, machining mechanisms, and applications to advanced materials [6,7]. Previous studies have shown that vibration amplitude, frequency, phase difference, trajectory shape, and the matching relationship between cutting speed and vibration velocity can all influence the instantaneous uncut chip thickness, contact state, shear angle, and interfacial force [8]. Therefore, UEVC should not be regarded simply as the superposition of a periodic displacement on conventional cutting. Instead [9], it dynamically regulates the material removal process by continuously altering the instantaneous tool velocity and engagement state over each vibration cycle [10].

The application of UEVC to the precision machining of tungsten and tungsten alloys has attracted increasing attention. Suzuki et al. applied elliptical vibration cutting to the machining of tungsten alloy molds for optical components and demonstrated that the method could alleviate severe tool wear, brittle fracture, and adhesion commonly encountered in conventional diamond cutting [11]. Pan et al. subsequently investigated surface roughness prediction and single-crystal diamond tool wear during UEVC of tungsten heavy alloys, showing that appropriately selected ultrasonic vibration parameters could improve surface quality and substantially modify tool wear behavior [12,13]. Bai et al. demonstrated that the synergistic coupling of ultrasonic elliptical vibration and cold plasma assistance could effectively improve the surface integrity of W-Mo70 alloys by reducing surface roughness, suppressing crack and tearing defects, and promoting more uniform material removal during precision turning [14]. Zhao et al. further examined the effects of different vibration directions and cutting trajectories and found that vibrations along the depth-of-cut and cutting speed directions exert different influences on chip formation, whereas a two-dimensional elliptical trajectory can markedly modify the strain rate, cutting force, and chip morphology [15]. In addition, the matching between system vibration characteristics and machining parameters may affect cutting chatter, surface morphology, and tool wear during UEVC, further indicating the strongly dynamic nature of the process [16].

In recent years, research on UEVC of tungsten alloys has gradually shifted from the simple evaluation of surface quality toward a deeper understanding of material removal behavior and microstructural evolution mechanisms. Yin et al. investigated the effects of different vibration directions through experiments and finite element simulations and found that UEVC can significantly alter the strain rate, cutting force, and plastic material removal behavior within the cutting zone [17]. Their subsequent study on phase difference further demonstrated that the shape of the elliptical trajectory affects tool–workpiece contact, cutting force, temperature, strain rate, and surface dislocation density [18]. Su et al. observed through cutting experiments using cemented carbide tools that ultrasonic impact can promote dislocation motion and subsurface grain refinement, while shifting the material removal mode toward a more ductile-dominated regime [19]. In addition, tungsten alloys exhibit pronounced differences in mechanical response under high-strain-rate conditions, and high-rate loading, local stress states, and ultrasonic scratching can all influence their plastic deformation and microstructural evolution [20,21,22]. These studies indicate that the effects of UEVC on tungsten cannot be adequately described solely by the average cutting force or final surface roughness; rather, its material removal mechanism should be understood from the perspectives of dynamic loading and microstructural evolution.

However, macroscopic cutting experiments and post-machining microstructural characterization generally provide only information on cutting forces, surface morphology, and the final subsurface structure, making it difficult to directly capture transient atomic motion and the nucleation, propagation, and reorganization of dislocations on timescales of tens of picoseconds or less. Molecular dynamics (MD) simulation enables direct tracking of the spatiotemporal evolution of individual atoms and has therefore become an important approach for investigating material removal and subsurface defect formation during nanoscale cutting. Wang et al. employed MD simulations to examine the influence of diamond tool geometry on the nano-cutting of single-crystal tungsten and showed that tool geometry can significantly affect cutting force, cutting-induced thermal response, subsurface damage, and the activity of 1/2⟨111⟩ and ⟨100⟩ dislocations [23]. Dong et al. further identified the minimum uncut chip thickness in tungsten nano-cutting and demonstrated that the chip formation state can alter the dislocation-dominated plastic removal mechanism [24]. These findings suggest that dislocation activity not only governs the atomic-scale plastic response of tungsten but is also closely associated with the material removal mode and the development of subsurface damage.

At the atomic scale, Wang et al. reported that the transient high shear stress generated during UEVC can activate pronounced dislocation activity, lattice distortion, and local structural reorganization [25]. Their subsequent study further demonstrated that the cyclic loading–unloading process in UEVC can alter the effective material removal scale and subsurface dislocation propagation behavior, indicating that transient mechanical responses must be considered when interpreting ultrasonic cutting mechanisms rather than relying solely on time-averaged quantities [26]. Studies combining scratching experiments with MD simulations have also shown that the high transient stresses induced by UEVC may activate more intensive slip activity, suggesting that ultrasonic vibration does not simply “reduce dislocations” throughout the entire cutting process but instead modifies the activation and evolution pathways of dislocations [27]. More recent work has proposed a micro-incremental cutting mechanism, in which high-frequency, small-scale periodic material removal introduces pre-straining within the deformation zone and regulates the subsequent shearing process [28]. Meanwhile, studies on crystallographic orientation have revealed that both instantaneous material removal and subsurface dislocation evolution during UEVC exhibit pronounced orientation dependence [29]. These findings further demonstrate that the dislocation response under ultrasonic-assisted cutting is inherently transient and path dependent.

It should be noted, however, that most existing atomic-scale studies have focused on single-crystal pure tungsten or tungsten heavy alloys such as W–Ni–Fe systems, whose compositions and microstructural characteristics differ substantially from those of W-Mo70 solid-solution alloys. Therefore, although these studies provide important references for understanding dislocation activity and material removal in BCC tungsten-based materials, their conclusions cannot be directly transferred to W-Mo70. Based on the scope of the available literature, a systematic cross-scale understanding of the intrinsic relationships among periodic loading, atomic-scale material removal, and transient dislocation evolution in W-Mo70 under conventional cutting and UEVC remains lacking. This assessment is derived from the current research coverage of tungsten and tungsten heavy alloy systems and does not imply that tungsten alloys with different compositions necessarily share identical deformation mechanisms.

In our previous work, conventional cutting, UEVC, CC cutting, and hybrid-assisted precision turning experiments were conducted on W–Mo70 alloys. The experimental results showed that, compared with conventional cutting, UEVC significantly modified both the cutting force response and the subsurface deformation state. Under conventional cutting, the cross-sectional microstructure exhibited a relatively pronounced plastically deformed layer, stacking faults, dislocation pile-ups, and transgranular microcracks, whereas the UEVC condition was characterized more prominently by vibration marks, lattice distortion, and transitional crystalline layers, indicating a distinct mode of structural reorganization. The previous experiments also demonstrated that the major cutting force components measured under UEVC were markedly lower than those obtained under conventional cutting. These observations provide both macroscopic and microstructural evidence for understanding the ultrasonic-assisted machining behavior of W-Mo70. However, post-machining SEM characterization cannot directly reveal how dislocations nucleate, evolve, and reorganize within an individual vibration cycle.

To address this limitation, the present study employs the previously published experimental results as a macroscopic benchmark and establishes molecular dynamics models of CC and UEVC for W-Mo70 alloys. It should be emphasized that the earlier experimental data are used only as previously reported evidence and as a reference for trend comparison, rather than being republished as new experimental results. The present work mainly compares CC and UEVC in terms of material removal behavior, cutting force response, periodic tool–workpiece loading, atomic kinetic response, and dislocation evolution identified by the dislocation extraction algorithm (DXA). In addition, the transient defect evolution revealed by MD simulations is correlated across scales with the subsurface microstructural characteristics observed experimentally. Particular attention is devoted to distinguishing the progressive accumulation of defects under continuous loading from the transient dislocation activation and defect reorganization associated with ultrasonic cyclic loading–unloading, thereby elucidating the atomic-scale mechanisms by which UEVC regulates material removal and subsurface deformation in W-Mo70 alloys.

In addition to our previous experimental results [28], several independent studies have reported similar effects of ultrasonic vibration-assisted cutting, including reduced cutting resistance, modified tool–workpiece contact behavior, and improved surface integrity [8,9,10,11]. These studies support that the beneficial effects of UEVC originate from periodic contact modulation and dynamic loading–unloading behavior rather than continuous mechanical interaction.

## 2. Experimental Design

### 2.1. Experimental Materials

The main physical properties of the W-Mo70 alloy used in the molecular dynamics simulations are summarized in Table 1.

Considering the high hardness and strong machining resistance of the W–Mo alloy, a synthetic monocrystalline diamond (MCD) tool was employed for the ultra-precision turning experiments. The tool model was D04250102, featuring a triangular insert with a side length of 9.6 mm and a thickness of 2.38 mm. The tool had a nose radius of 1.2 mm, a rake angle of 0°, and a clearance angle of 15°. Owing to its high hardness, sharp cutting edge, and excellent dimensional stability, the MCD tool is well suited to the stringent requirements for tool geometry accuracy and cutting stability during ultra-precision turning of W–Mo70 alloys. The tool type and geometric parameters adopted in the previously reported experiments were consistent with those described above.

As shown in Table 2, to facilitate the correlation between the experimental results and the subsequent atomic-scale simulations, a BCC random solid-solution model was constructed in the MD simulations using the same W/Mo atomic composition as that of the experimental alloy, while a diamond crystal structure was employed for the cutting tool. It should be noted that the experimentally used tool nose radius and cutting dimensions cannot be directly scaled to the nanoscale MD model in a geometrically proportional manner. Therefore, the experimental material and tool information was primarily used to maintain consistency in the material system and the underlying cutting physics, rather than to establish a direct geometric correspondence between the macroscopic experimental configuration and the atomistic model.

**Figure 1:**
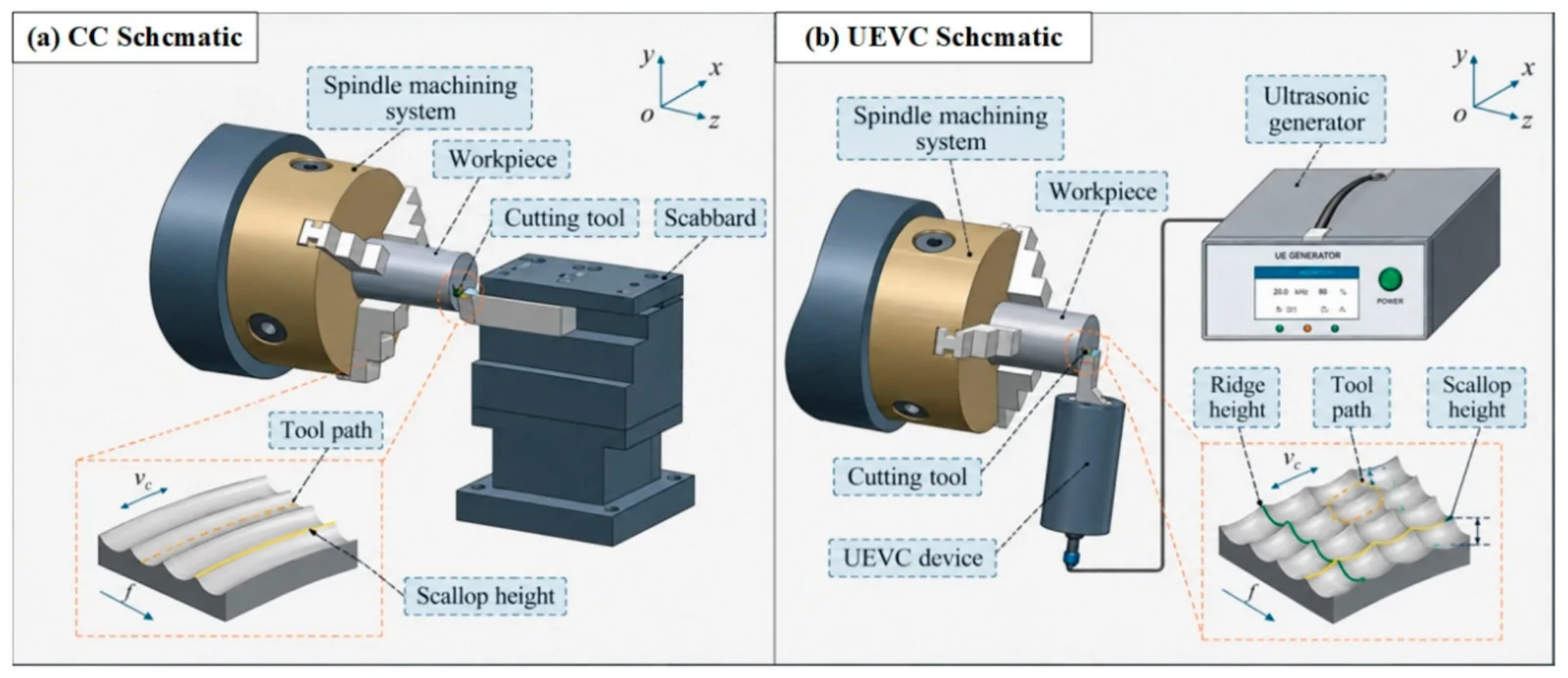
Schematic illustrations of CC and UEVC in ultra-precision end-face turning of W-Mo70 alloys: (**a**) conventional cutting configuration, showing the spindle machining system, workpiece, cutting tool, tool path, and resulting scallop profile; (**b**) UEVC configuration, showing the ultrasonic vibration device and generator, elliptical tool trajectory, and characteristic ridge–scallop surface morphology induced by periodic tool motion.

As shown in Figure 1, the ultra-precision turning experiments were conducted on a UPT250 ultra-precision machine tool. The machine provides travel ranges of 120 and 190 mm along the X- and Z-axes, respectively, with a positioning accuracy of 0.3 μm and a maximum spindle speed of 3000 r/min. Ultrasonic vibration was generated using a TAGA EL-50Σ ultrasonic system operating at a frequency of 20 ± 2 kHz. The longitudinal and bending vibration amplitudes could each be adjusted within the range of 0–12 μm. A phase difference of π/2 was imposed between the two orthogonal vibration components, thereby producing a two-dimensional elliptical trajectory.

In the previously reported experiments, different elliptical vibration trajectories were generated by independently adjusting the amplitudes of the two orthogonal vibration components. The complete experimental scheme included conventional cutting and UEVC, together with an investigation of the effects of different vibration amplitude ratios on the machining response. However, the present molecular dynamics study focuses exclusively on the influence of mechanical vibration on material removal. Therefore, only the conventional cutting and UEVC conditions, which allow for a direct comparison, were selected from the previous experiments as macroscopic benchmarks.

For consistency with the notation used in the MD simulations, the ordinary cutting condition denoted as OC in the previously published experimental study is referred to as conventional cutting (CC) in the present work. The experimental conditions used for the direct comparison between CC and UEVC employed identical basic cutting parameters, including a spindle speed of 150 r/min, a feed rate of 2 mm/min, and a depth of cut of 5 μm. Under the UEVC condition, the ultrasonic elliptical vibration amplitude ratio was set to λ = 1:1. Since the spindle speed, feed rate, and depth of cut were maintained identical for the CC and UEVC conditions, the observed differences between the two machining modes were primarily associated with the introduction of ultrasonic elliptical vibration.

### 2.2. Molecular Dynamics Model and Simulation Setup

To further elucidate the atomic-scale mechanisms governing material removal and defect evolution in W-Mo70 alloy during conventional cutting and ultrasonic elliptical vibration-assisted cutting, three-dimensional molecular dynamics (MD) models were established using LAMMPS [30]. Separate models were constructed for CC and UEVC. Except for the prescribed tool motion, the two models employed identical workpiece dimensions, initial crystal structures, boundary conditions, interatomic potentials, and thermal treatment procedures, thereby ensuring direct comparability between the two cutting conditions. In particular, the UEVC model retained the same workpiece geometry, tool geometry, interaction potentials, and boundary conditions as those used in the CC model, with the tool trajectory being the only fundamental difference between the two simulations.

#### 2.2.1. Atomic Model and Boundary Conditions

The W-Mo70 workpiece was constructed using a body-centered cubic (BCC) lattice with an initial lattice constant of 3.15246 Å. A pure W lattice was first generated, after which 70 at.% of the W atoms were randomly replaced by Mo atoms, resulting in a random substitutional solid solution containing 30 at.% W and 70 at.% Mo. Considering the stochastic nature of atomic arrangement in random solid solutions, additional simulations with independent atomic configurations were conducted to examine the influence of atomic randomness to evaluate the influence of structural randomness on the cutting response.

The dimensions of the workpiece along the x-, y-, and z-directions were approximately 15.00, 9.96, and 10.13 nm, respectively. The x-direction was defined as the primary cutting direction, the y-direction as the depth-of-cut or normal direction, and the z-direction as the model thickness direction. Non-periodic boundary conditions were applied along the x- and y-directions, while a periodic boundary condition was imposed along the z-direction. Considering the intrinsic limitation of atomistic simulations, the present model size was selected as a compromise between computational efficiency and the capability of capturing the dominant deformation region. Although a larger model size may further reduce possible finite-size effects, the present model provides sufficient atomic resolution to compare the relative deformation behavior between conventional cutting and ultrasonic elliptical vibration cutting. Non-periodic boundary conditions were applied along the x- and y-directions, while a periodic boundary condition was imposed along the z-direction, i.e.,

boundary = *f*, *f*, *p*. (1)

where *f* denotes the fixed-atom region, and *p* represents the thermostatted atom region for NVT temperature control.

This boundary condition configuration preserves the free machining surface while employing periodicity along the z-direction to approximate a cutting state that extends continuously along the tool width direction. To minimize artificial boundary constraints, the fixed boundary layer and thermostatic boundary layer were placed away from the primary cutting zone, while the deformable mobile region was retained as the main area for chip formation and plastic deformation. The same spatial partitioning of the fixed, thermostatic, and mobile regions was adopted for both CC and UEVC simulations to ensure direct comparison between the two cutting conditions. The bottom of the workpiece and the side region away from the primary cutting zone were assigned as fixed layers to maintain the overall stability of the substrate. A thermostatic layer was placed adjacent to the fixed layer to absorb part of the energy transferred into the workpiece during cutting, while the remaining atoms constituted the mobile region in which material removal and plastic deformation primarily occurred. The same spatial partitioning of the fixed, thermostatic, and mobile regions was adopted for both cutting models. The cutting tool was constructed using a diamond crystal structure, with the C atoms generated according to a diamond lattice. The tool tip contained a rounded cutting edge with an atomic-scale edge radius of approximately 1.43 nm. During the simulations, the relative positions of the atoms within the diamond tool were maintained rigid, thereby eliminating additional effects associated with tool deformation and enabling the deformation behavior of the W-Mo70 workpiece to be analyzed more clearly. To facilitate the reading and verification of the simulation configuration, all key parameters of the MD models are summarized in Table 3.

**Figure 2:**
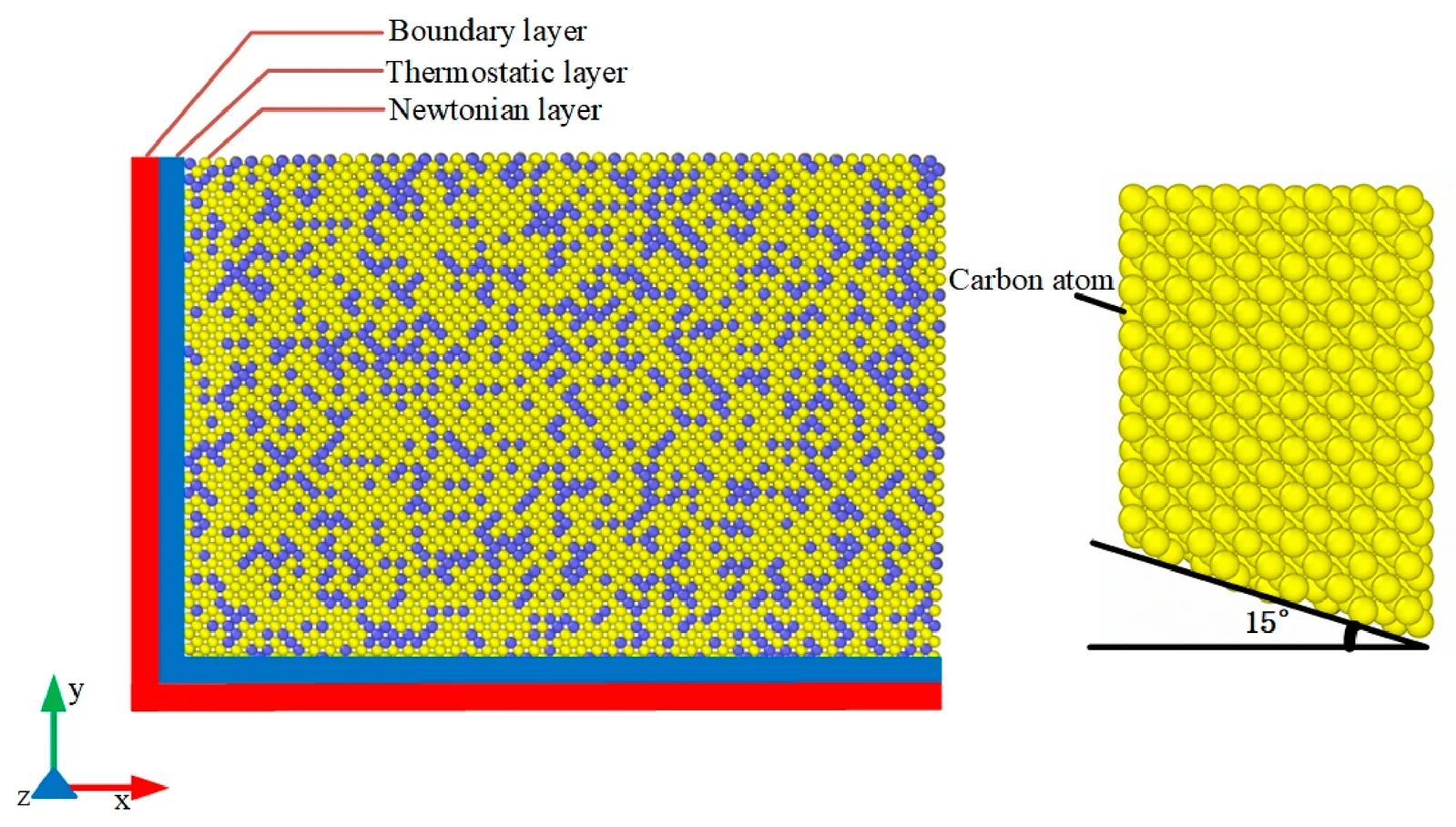
The atomic configuration of the three-dimensional MD ultrasonic cutting model for the W–Mo alloy, showing the coordinate system, three-layer workpiece structure (fixed boundary layer, thermostatic layer, and Newtonian layer), diamond cutting tool, cutting direction, and tool geometry.

The initial atomic configuration of the three-dimensional MD ultrasonic cutting model for the W-Mo alloy is shown in Figure 2. The x-axis is defined as the primary cutting direction, the y-axis corresponds to the depth-of-cut (normal) direction, and the z-axis represents the thickness direction of the simulation model. As illustrated in the figure, the workpiece is divided into three functional regions: the bottom-most fixed boundary layer whose atoms remain stationary to prevent overall rigid displacement; the middle thermostatic layer controlled by the NVT ensemble to dissipate cutting-induced heat; and the upper deformable Newton layer, where atoms follow Newton’s equations of motion to reproduce material deformation and chip formation. A diamond cutting tool with a fixed rake angle is placed on the right side of the workpiece for ultrasonic cutting simulation.

#### 2.2.2. Interatomic Potentials

The interatomic interactions directly determine the elastic–plastic response, atomic bonding, and tool–workpiece interfacial behavior in MD simulations. Therefore, appropriate interatomic potentials were employed to describe the C–C, W–W/Mo–Mo/W–Mo, and C–W/C–Mo interactions, respectively. The W–W, Mo–Mo, and W–Mo interactions within the W-Mo70 alloy were described using an EAM/FS-type binary W–Mo potential [31]. The C–C interactions within the diamond cutting tool were represented by the Tersoff potential [32]. The interactions between the diamond tool and the W-Mo70 workpiece were described using the Lennard–Jones (LJ) potential [33]. The LJ parameters for C–W and C–Mo interactions were set as ε = 0.04 eV and σ = 3.0 Å, respectively. These parameters have been widely adopted in previous molecular dynamics simulations of diamond-based cutting processes to describe weak van der Waals interactions between carbon tools and metallic substrates [34]. Since the present study focuses on the comparative deformation mechanisms between CC and UEVC rather than the exact reproduction of experimental cutting force magnitudes, the same LJ parameters were consistently applied to both cutting conditions [35].

A cutoff distance of 10 Å was employed. The corresponding Lennard–Jones potential can be expressed as follows:

(2) $$E_{\mathrm{ij}\;}=\;4\varepsilon \left[{\left(\frac{\sigma }{r_{\mathrm{ij}}}\right)}^{12}-{\left(\frac{\sigma }{r_{\mathrm{ij}}}\right)}^{6}\right]$$

where r denotes the separation distance between a C atom in the cutting tool and a W or Mo atom in the workpiece. In the present simulation, identical LJ parameters were employed for the C–W and C–Mo interactions.

#### 2.2.3. Conventional and Ultrasonic Tool Motions

Under conventional cutting conditions, the diamond tool moved at a constant velocity along the negative x-direction, and the nominal cutting speed was set as follows:

(3) $$V_{c}\;=\;2.5\;Å/\mathrm{ps}\;=\;250\;m/s$$

This velocity is substantially higher than the actual linear cutting speed used in macroscopic ultra-precision turning experiments, which is necessitated by the limited timescale accessible to molecular dynamics simulations. For the experimental conditions adopted in the direct CC–UEVC comparison (spindle speed *n* = 150 r/min, workpiece diameter *D* = 25 mm), the actual linear cutting speed is as follows:

(4) $$V_{exp}=\frac{\pi DN}{60}=0.00327\;m/s$$

The nominal MD cutting speed (250 m/s) is therefore approximately 7.6 × 10^4^ times higher than the experimental value, corresponding to a difference of nearly five orders of magnitude. This substantial mismatch in velocity scale, together with differences in strain rate, model size, and tool geometry, indicates that the present MD simulations are mainly used to reveal the relative effects and atomic-scale mechanisms of CC and UEVC rather than to quantitatively reproduce the experimental cutting force magnitude. The relatively high cutting speed enables a sufficient material removal distance to be achieved within several tens of picoseconds. Therefore, the present MD simulations are primarily used to compare the relative responses of CC and UEVC and to elucidate their atomic-scale mechanisms, rather than to directly reproduce the experimental cutting speed. In the UEVC model, periodic vibrations along the x- and y-directions were superimposed on the same constant feed motion. In the present model, the vibration amplitudes in the two orthogonal directions were set as follows:

(5) $$A_{x}\;={\;A}_{y}\;=\;15\;Å\;=\;1.5\;\mathrm{nm}$$

The vibration frequency was set as follows:

(6) $$f\;=\;0.02\;{\mathrm{ps}}^{-1}\;=\;20\;\mathrm{GHz}$$

The corresponding vibration period was 50 ps. All of the above parameters were consistent with those implemented in the UEVC simulation. The tool displacement was defined as follows:

(7) $$x\left(t\right)\;=\;-{\;V}_{c}t\;+\;A_{x}\left[\cos\left(\omega t\right)-\;1\right]$$

(8) $$v_{y}\left(t\right)=A_{y}\omega \cos\left(\omega t\right)$$

where:

(9) ω=2πf

The corresponding instantaneous velocities were expressed as follows:

(10) $$v_{x}(t)=-V_{c}-A_{x}\omega \sin(\omega t)$$

This motion equation directly corresponds to the definitions of tool displacement and velocity implemented in the current LAMMPS model. Over one complete vibration cycle, the nominal feed distance of the tool was approximately

(11) $$V_{c}T=12.5\;\mathrm{nm}.$$

Accordingly, the UEVC model was able to capture a complete loading–unloading–reloading sequence within one vibration cycle, providing the basis for subsequent analysis of the normal-force response and transient dislocation evolution.

(12) $$d_{\mathrm{cycle}\;}={\;V}_{c}T=12.5\;\mathrm{nm}$$

(13) $$\lambda =\frac{A_{x}}{A_{y}}=1$$

(14) $$f_{\exp}=20\;\pm \;2\;\mathrm{kHz}$$

All models were first subjected to energy minimization using the FIRE algorithm to eliminate any unphysical local high-energy configurations that may have arisen during initial model construction. Subsequently, the initial temperature of the W–Mo workpiece was set to 300 K, followed by a 20 ps thermal relaxation process to bring the system to a relatively stable initial state. An integration time step of 1 fs was employed in the present simulations, i.e.,

(15) $$N_{\mathrm{relax}}=20,000.$$

The energy minimization procedure, the initial velocity assignment at 300 K, and the integration time-step setting were all consistent with those implemented in the simulation program. The thermal relaxation stage was performed for a total of 20,000 time steps, corresponding to 20 ps.

During the production cutting stage, the direct Langevin thermostat applied to the mobile workpiece region was removed, while the thermostatic boundary region continued to act as a heat bath. This treatment minimizes artificial interference with the atomic dynamics in the primary cutting zone. In the current simulation program, the Langevin thermostat acting on the mobile region was indeed removed prior to the onset of cutting.

The cutting forces were obtained by summing the atomic forces acting on all C atoms of the diamond tool along the x-, y-, and z-directions. Here, Fx was defined as the main cutting force, Fy as the normal force, and Fz as the transverse force along the periodic thickness direction. The total tool force components in all three directions were calculated using the reduce sum operation in LAMMPS.

To maintain a consistent horizontal coordinate for CC and UEVC, the nominal cutting distance corresponding to the constant feed velocity was used as the horizontal axis for result analysis, rather than the instantaneous tool displacement in the UEVC model. The nominal cutting distance was defined as follows:

(16) $$d_{\mathrm{nom}}\;=\;V_{c}t$$

The cutting force and the calculated kinetic temperature were output every 2000 time steps, such that two adjacent data points corresponded to a nominal cutting distance increment of approximately 0.5 nm. The calculated kinetic temperature of the mobile workpiece region was obtained using the compute temp command in LAMMPS. This quantity reflects the overall kinetic energy response of the mobile workpiece atoms; however, it may also contain contributions from the directional motion of the chip and the ordered velocity components induced by the periodic UEVC motion. Therefore, it is referred to in this study as the calculated kinetic temperature and is used primarily to compare the atomic kinetic responses under the two cutting conditions, rather than being directly interpreted as a rigorous local thermodynamic cutting temperature. The temperature calculation domain and time-step settings were fully consistent with those implemented in the simulation program.

The UEVC tool trajectory was output every 200 time steps, including the nominal cutting distance, simulation time, tool displacements in the x- and y-directions, and the corresponding instantaneous velocities. These data were used to analyze the coupling relationship between the vibration phase of the tool and the normal-force response. The production cutting stage of the UEVC simulation was performed for a total of 57,500 time steps, corresponding to 57.5 ps, or approximately 1.15 ultrasonic vibration cycles. The UEVC production-cutting stage consists of 57,500 timesteps, corresponding to approximately 1.15 vibration cycles at the prescribed frequency of 20 GHz. Due to the high computational cost of atomistic simulations, the present simulation window was selected to capture the representative loading–unloading process within a vibration cycle rather than to provide long-term statistical sampling over multiple cycles.

The post-cutting atomic configurations were visualized and analyzed using OVITO [33]. Dislocation structures were extracted using the Dislocation Extraction Algorithm (DXA) [34], with the BCC lattice adopted as the reference crystal structure. To ensure direct comparability between CC and UEVC, identical DXA parameters were used for both cutting conditions, and atomic configurations at nominal cutting distances of 4, 8, 12, and 14 nm were selected for comparison. The total dislocation length and Burgers vector types were used to quantitatively characterize the accumulation and reorganization of lattice defects under different cutting modes.

## 3. Results and Discussion

### 3.1. Experimental Reference and MD Model Validation

To evaluate whether the molecular dynamics model can reasonably reproduce the mechanical response of the W-Mo70 alloy during ultra-precision cutting, the simulation results were first compared with the previously published experimental results in terms of overall trends. The previous ultra-precision turning experiments showed that, under conventional cutting conditions, the cutting force components were approximately Fx = 3.0 N and Fz = 3.6 N. After the introduction of ultrasonic elliptical vibration, the corresponding force components under UEVC decreased to approximately 1.2 N and 0.7 N, representing reductions of about 60% and 80.6%, respectively. These experimental force reductions are presented only as macroscopic evidence of the effectiveness of UEVC. Due to the substantial differences in cutting velocity and strain rate between experiments and MD simulations, the reduction ratios obtained from the two approaches are not directly comparable in magnitude.

These experimental results demonstrate that ultrasonic vibration can significantly modify the tool–workpiece interfacial loading state and reduce the overall cutting resistance.

The current MD simulations exhibited the same overall trend. Over one complete nominal vibration cycle, the cycle-averaged main cutting force under conventional cutting was ${\bar{F}}_{c,\mathrm{CC}}\;=\;332.7\;\mathrm{eV}/Å\;\approx \;533\;\mathrm{nN}$.

Meanwhile, under UEVC, the cycle-averaged main cutting force decreased to ${\bar{F}}_{c,\mathrm{UEVC}}\;=\;299.6\;\mathrm{eV}/Å\;\approx \;480\;\mathrm{nN}$.

Therefore, based on the present MD trajectory, the reduction in the cycle-averaged main cutting force induced by ultrasonic vibration was estimated as follows:

(17) $$\eta_{F_{c}}\;=\;\frac{{\bar{F}}_{c,\mathrm{CC}}\;-\;{\bar{F}}_{c,\mathrm{UEVC}}}{{\bar{F}}_{c,\mathrm{CC}}}\;\times \;100\%\;\approx \;9.9\%$$

This value represents the force reduction trend obtained from the current atomic configuration rather than an exact universal reduction ratio, because the random substitutional distribution of W and Mo atoms and thermal fluctuations may introduce statistical variations among independent MD trajectories.

### 3.2. Material Removal Characteristics Under CC and UEVC

**Figure 3:**
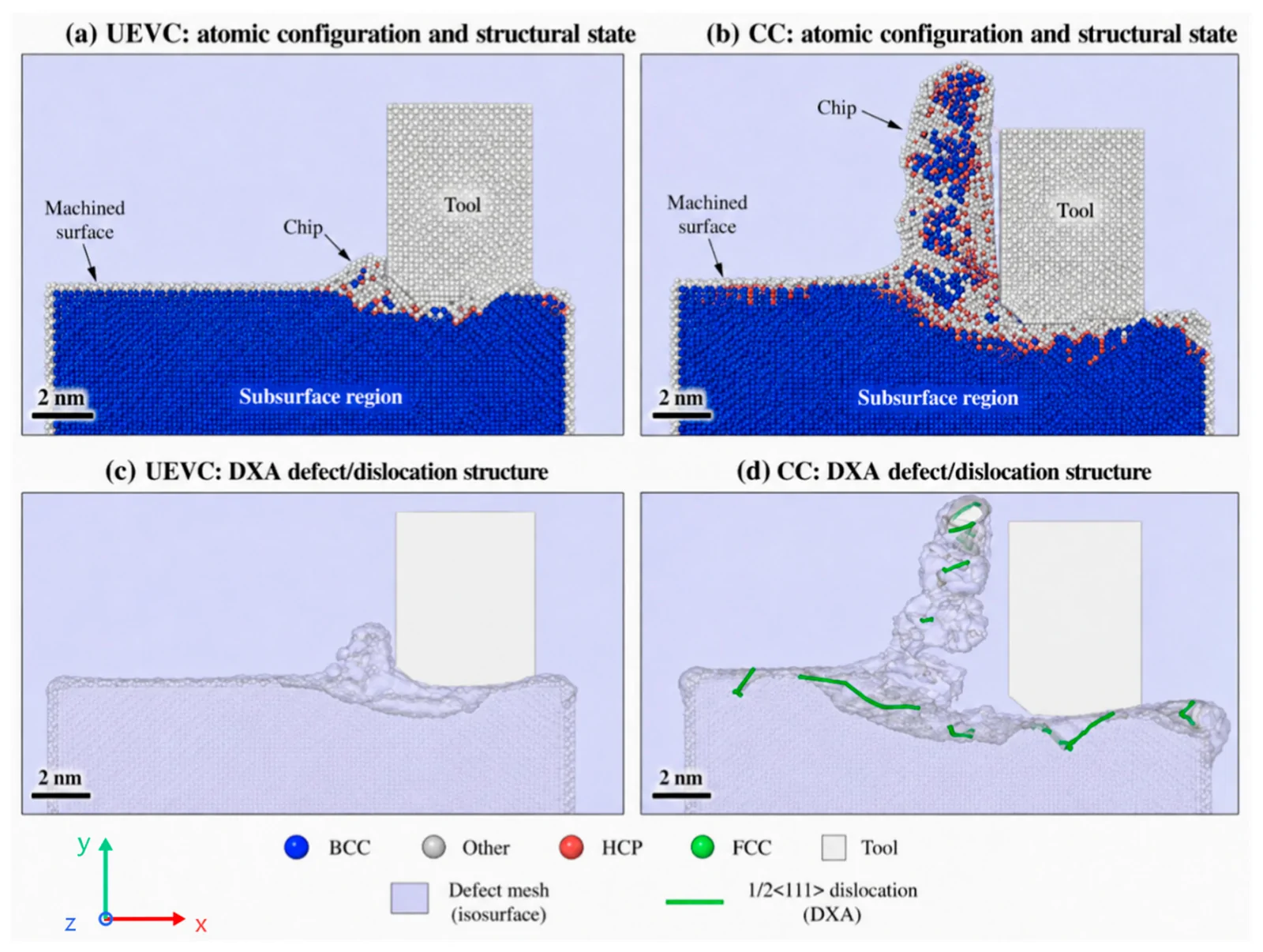
(**a**) UEVC: atomic configuration and structural state; (**b**) CC: atomic configuration and structural state; (**c**) UEVC: DXA defect/dislocation structure; (**d**) CC: DXA defect/dislocation structure. The micro-atomic microstructure and dislocation evolution of UEVC and CC in the middle cutting stage. In panels (**c**,**d**), the diamond cutting tool is depicted as a simplified white rectangular profile to avoid visual occlusion of intra-workpiece dislocation structures and subsurface defects by dense carbon atoms, enabling clear observation of atomic-scale deformation features.

**Figure 4:**
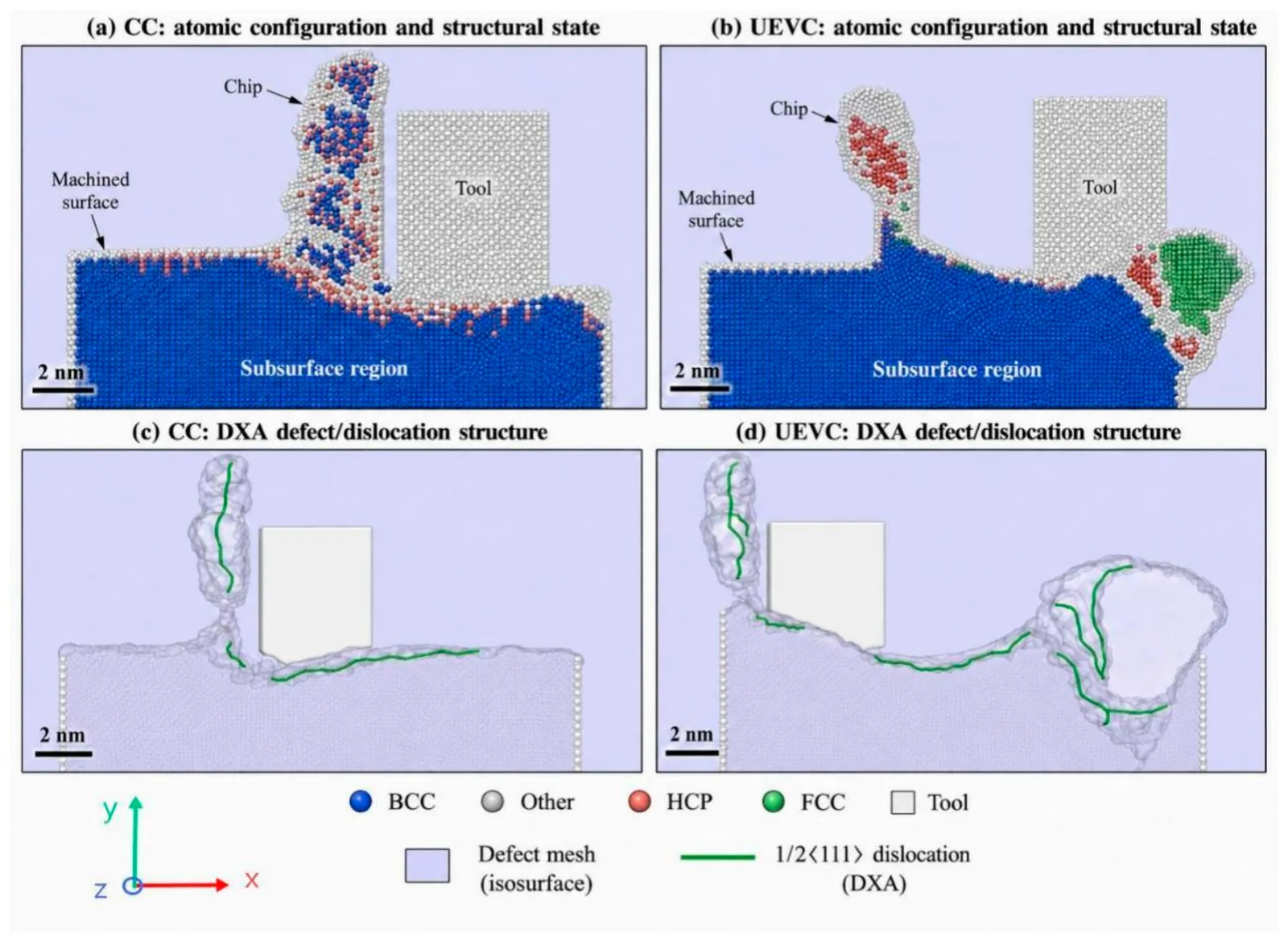
(**a**) CC: atomic configuration and structural state; (**b**) UEVC: atomic configuration and structural state; (**c**) CC: DXA defect/dislocation structure; (**d**) UEVC: DXA defect/dislocation structure. Micro-atomic structure and dislocation defects of CC and UEVC at final cutting distance.

Figure 3 and Figure 4 illustrate the evolution of the atomic configurations under CC and UEVC at the same nominal cutting distances. During conventional cutting, continuous mechanical interaction is maintained between the tool and the workpiece, causing the material ahead of the cutting edge to undergo sustained compressive and shear deformation. A large number of atoms migrate upward along the rake face and gradually form a relatively continuous chip structure. As the cutting distance increases, material accumulation ahead of the cutting edge and structural disturbance near the machined surface continue to develop, indicating that the material removal process is predominantly governed by continuous loading.

In contrast, during UEVC, a periodic vibration component in the normal direction is superimposed on the continuous feed motion, causing the instantaneous engagement state between the tool and workpiece to vary dynamically. In the simulation, the ultrasonic vibration amplitude was approximately 1.5 nm and the vibration frequency was set to 20 GHz, corresponding to a vibration period of approximately 50 ps. At a nominal cutting speed of 250 m/s, one complete vibration cycle corresponds to a nominal cutting distance of approximately 12.5 nm. Consequently, within each ultrasonic vibration cycle, the workpiece material experiences distinct loading, peak loading, and unloading stages.

Analysis of the atomic configurations shows that chip formation under CC is dominated by a continuous accumulation process, whereas under UEVC, both the material removal zone and chip morphology vary markedly with the vibration phase of the tool. This indicates that ultrasonic vibration does not merely introduce a small perturbation to the conventional cutting trajectory; instead, it periodically modulates the local mechanical state around the cutting edge and alters the trajectories of atomic motion in the deformation zone.

It is worth noting that the trajectory and force data obtained from the current simulations clearly demonstrate a periodic loading–unloading behavior. However, complete physical separation between the tool and workpiece throughout the vibration cycle has not yet been verified based on either the number of tool–workpiece contact atoms or the minimum interfacial distance. Therefore, in the MD analysis of the present study, the term “periodic loading–unloading” is considered more rigorous than directly defining the process as fully intermittent cutting.

This material removal mode provides the structural basis for the subsequent periodic responses of cutting force and defect evolution. Continuous cutting tends to promote the progressive accumulation of strain and lattice defects, whereas ultrasonic vibration continuously modulates the local load-bearing state of the material, thereby imparting a more pronounced time-dependent character to the plastic deformation process.

### 3.3. Cutting Force and Kinetic Response

**Figure 5:**
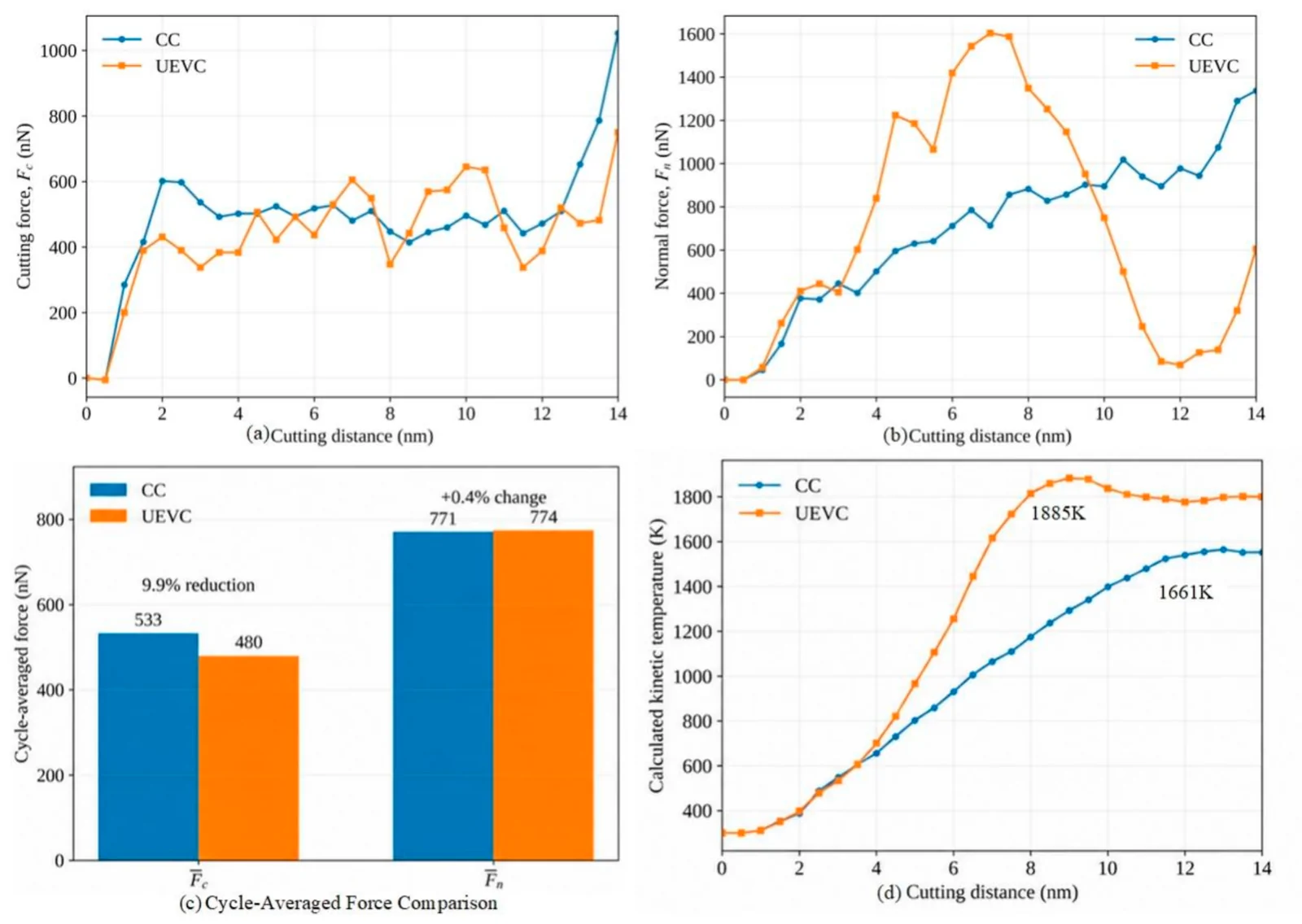
(**a**) Instantaneous cutting force; (**b**) instantaneous normal force; (**c**) cycle-averaged cutting and normal forces; and (**d**) calculated kinetic temperature in cutting region.

Figure 5 compares the variations in the main cutting force, normal force, and calculated kinetic temperature of the mobile workpiece region with nominal cutting distance under CC and UEVC conditions.

Regarding the main cutting force, both cutting conditions exhibited pronounced fluctuations after the tool entered the relatively stable material removal stage. However, the cycle-averaged main cutting force under UEVC was lower than that under CC. Considering the stochastic nature of the W-Mo70 random solid-solution structure, additional repeated simulations were performed to evaluate the possible influence of atomic configuration randomness. The observed force reduction represents the overall trend rather than an exact universal value. This result indicates that ultrasonic vibration can effectively reduce the average mechanical resistance in the cutting direction, which is qualitatively consistent with the reduction trend observed in the previously reported UEVC experiments. However, the absolute reduction magnitude differs because of the intrinsic mismatch between the experimental and MD conditions.

Compared with the main cutting force, the normal force exhibited a much more pronounced vibration-dependent response. Under CC, the normal force generally increased with cutting distance, reflecting the progressive buildup of compressive loading under continuous tool–workpiece interaction. In contrast, the normal force under UEVC underwent a distinct increase–peak–rapid decrease–increase sequence within the observed vibration cycle, exhibiting a characteristic loading–unloading response associated with ultrasonic vibration. Although the cycle-averaged normal forces under CC and UEVC were approximately 771 and 774 nN, respectively, indicating only a marginal change in the mean value, their instantaneous loading histories differed substantially. This demonstrates that the effect of ultrasonic vibration is not simply to reduce the average force in all directions, but rather to redistribute the normal load temporally over the vibration cycle.

The calculated temperature response also showed marked differences between the two cutting conditions. The calculated kinetic temperature response showed differences between the two cutting conditions. The maximum calculated kinetic temperature under CC was approximately 1661 K, whereas that under UEVC reached approximately 1885 K. Both CC and UEVC simulations were initially equilibrated at 300 K, which is also reflected by the starting point of the temperature curves in Figure 4d. This higher value reflects an increased atomic kinetic energy contribution under ultrasonic vibration, including both thermal fluctuations and vibration-induced directional atomic motion. However, the temperature quantity used in this study was calculated from the total kinetic energy of the mobile workpiece atoms and did not fully exclude the contributions of directed cutting flow and ordered velocity components induced by ultrasonic vibration. Therefore, it is more appropriately interpreted as the calculated kinetic temperature, rather than being directly regarded as the actual thermodynamic cutting temperature measured in experiments.

### 3.4. Periodic Tool–Workpiece Loading Behavior

**Figure 6:**
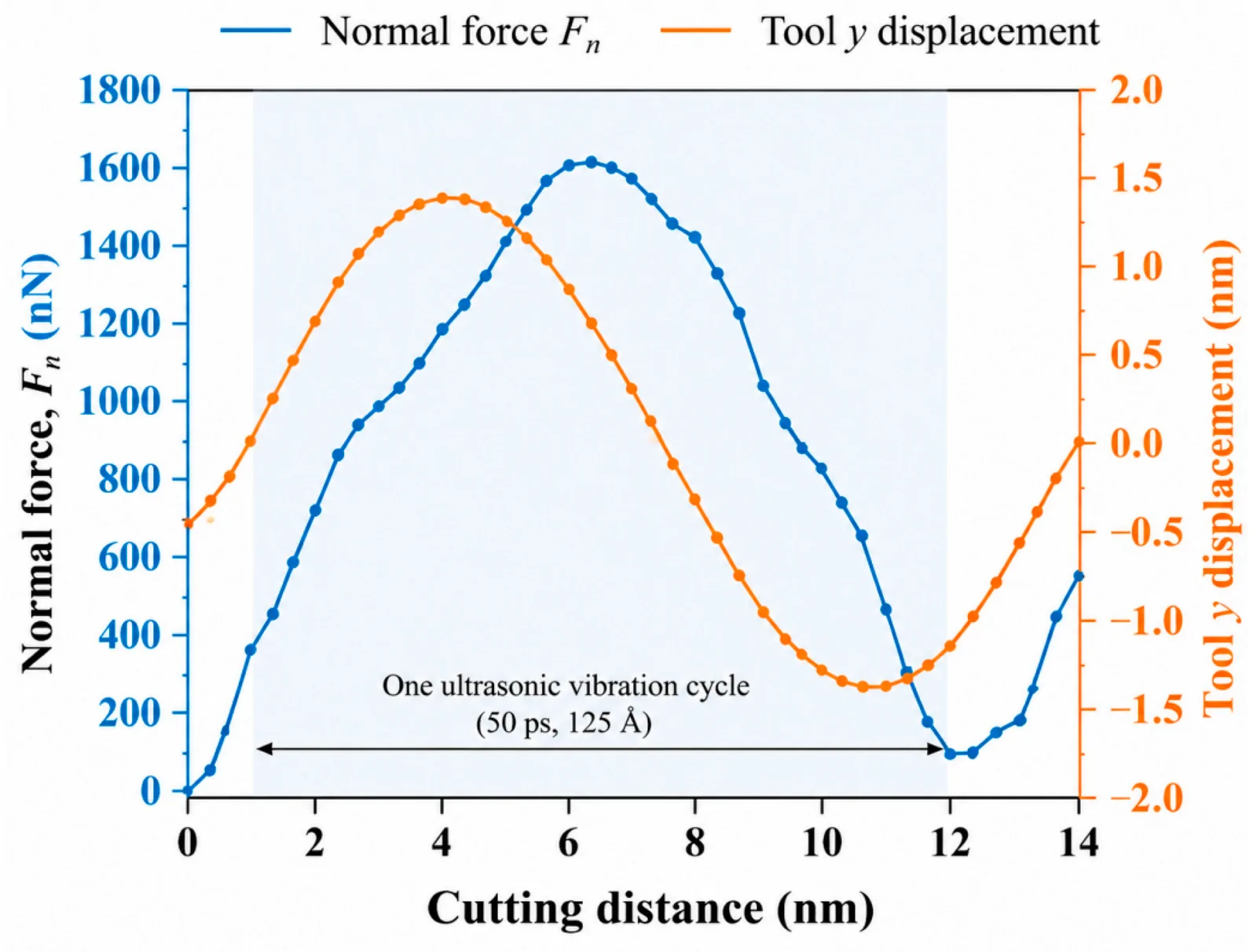
The coupling between the normal force and tool displacement in the y-direction during UEVC over one ultrasonic vibration cycle.

To further clarify the relationship between the variation in normal force and the ultrasonic vibration motion of the tool during UEVC, tool displacement in the y-direction was analyzed synchronously with the normal force, as shown in Figure 6. As the tool entered the normal loading stage, the tool–workpiece interaction gradually intensified and the normal force increased rapidly, reaching a high-load state of approximately 1600 nN at a nominal cutting distance of about 7 nm. Subsequently, the normal motion of the tool reversed and entered the retraction stage, during which the normal force decreased sharply. At a nominal cutting distance of approximately 12 nm, the normal force decreased to about 69 nN.

The relationship between the tool displacement and normal force exhibits a hysteretic behavior during UEVC. The instantaneous normal force is not determined solely by the tool position, but also depends on the loading–unloading history and the evolving tool–workpiece interaction state. Multiple factors, including chip accumulation, local contact area, prior plastic deformation history, and atomic-scale structural rearrangement near the cutting edge, contribute to the observed force variation.

From a mechanistic perspective, the periodic variation in load can be attributed to the cyclic modulation of the tool–workpiece interfacial loading state by ultrasonic vibration. As the tool penetrates into the workpiece, a high-load contact state is progressively established, whereas the interfacial interaction weakens substantially during the tool-retraction stage. With the onset of the subsequent vibration cycle, the tool enters the loading stage again. Consequently, the tool–workpiece interaction under UEVC exhibits a repeated loading–unloading–reloading sequence.

The previously reported experiments also demonstrated that ultrasonic elliptical vibration reduces the effective contact area and sustained interfacial friction through periodic dynamic contact, thereby lowering the macroscopic cutting force. The present molecular dynamics results further reveal that, at the atomic scale, this macroscopic force reduction behavior corresponds to a pronounced periodic loading–unloading process rather than a simple state of continuously low interfacial loading.

### 3.5. Dislocation Evolution and Structural Reorganization

The Dislocation Extraction Algorithm (DXA) was employed to analyze dislocation evolution during CC and UEVC. Since the W–Mo matrix is a body-centered cubic (BCC) solid solution, the BCC lattice was adopted as the reference crystal structure for both cutting conditions, and identical DXA parameters were maintained throughout the analysis. To ensure direct comparability, atomic configurations at nominal cutting distances of 4, 8, 12, and 14 nm were selected for comparison.

As observed from the dynamic dislocation evolution process in Figure 3, the periodic unloading effect during UEVC continuously promotes dislocation annihilation and rearrangement in the subsurface region throughout the cutting process. Consistent with this evolution trend, the cross-sectional residual defect morphology in Figure 4 shows that the subsurface residual defect layer under UEVC is noticeably thinner than that in CC. Quantitative statistics further indicate that the residual dislocation density in the UEVC subsurface region is 32% lower than that under CC, which verifies the defect softening effect induced by periodic ultrasonic loading. This result is also consistent with the cutting force reduction trend presented in Section 3.1. Under CC conditions, the total length of identifiable dislocations exhibited an overall increasing trend with cutting distance. At a nominal cutting distance of 4 nm, the total dislocation length was approximately 1.47 nm. As the cutting distance increased to 8 and 12 nm, the total dislocation length increased to approximately 2.27 and 2.55 nm, respectively, and further increased to approximately 3.09 nm at 14 nm. Throughout the analyzed cutting interval, all identified dislocations belonged to the 1/2⟨111⟩ Burgers vector family, while no pronounced ⟨100⟩ or other dislocation types were detected.

These results indicate that during conventional cutting, the continuously strong mechanical interaction between the tool and workpiece promotes the progressive accumulation of local plastic strain and lattice defects. Although the number of identified dislocation segments did not increase strictly monotonically at every cutting stage, the total dislocation length exhibited a persistent upward trend. This suggests that dislocation evolution involves not only the nucleation of new dislocations, but also the extension, connection, and structural rearrangement of existing dislocation lines. Therefore, compared with simply counting the number of dislocation segments, the total dislocation length provides a more meaningful indicator of defect evolution within the present simulation framework. However, the absolute dislocation length values should be interpreted with caution because they may be affected by atomic-scale statistical fluctuations associated with the random solid-solution configuration and thermal evolution.

In contrast, dislocation evolution under UEVC exhibited pronounced transient characteristics. At a cutting distance of 4 nm, no complete DXA-identifiable dislocation lines were detected. When the cutting distance reached 8 nm, three dislocation segments appeared transiently, with a total length of approximately 3.35 nm, which was greater than the corresponding value of approximately 2.27 nm under CC. This result indicates that ultrasonic vibration does not continuously suppress dislocation nucleation throughout the cutting process. Instead, during stages of relatively high instantaneous loading, strong local lattice shear and pronounced dislocation activation may still occur.

**Figure 7:**
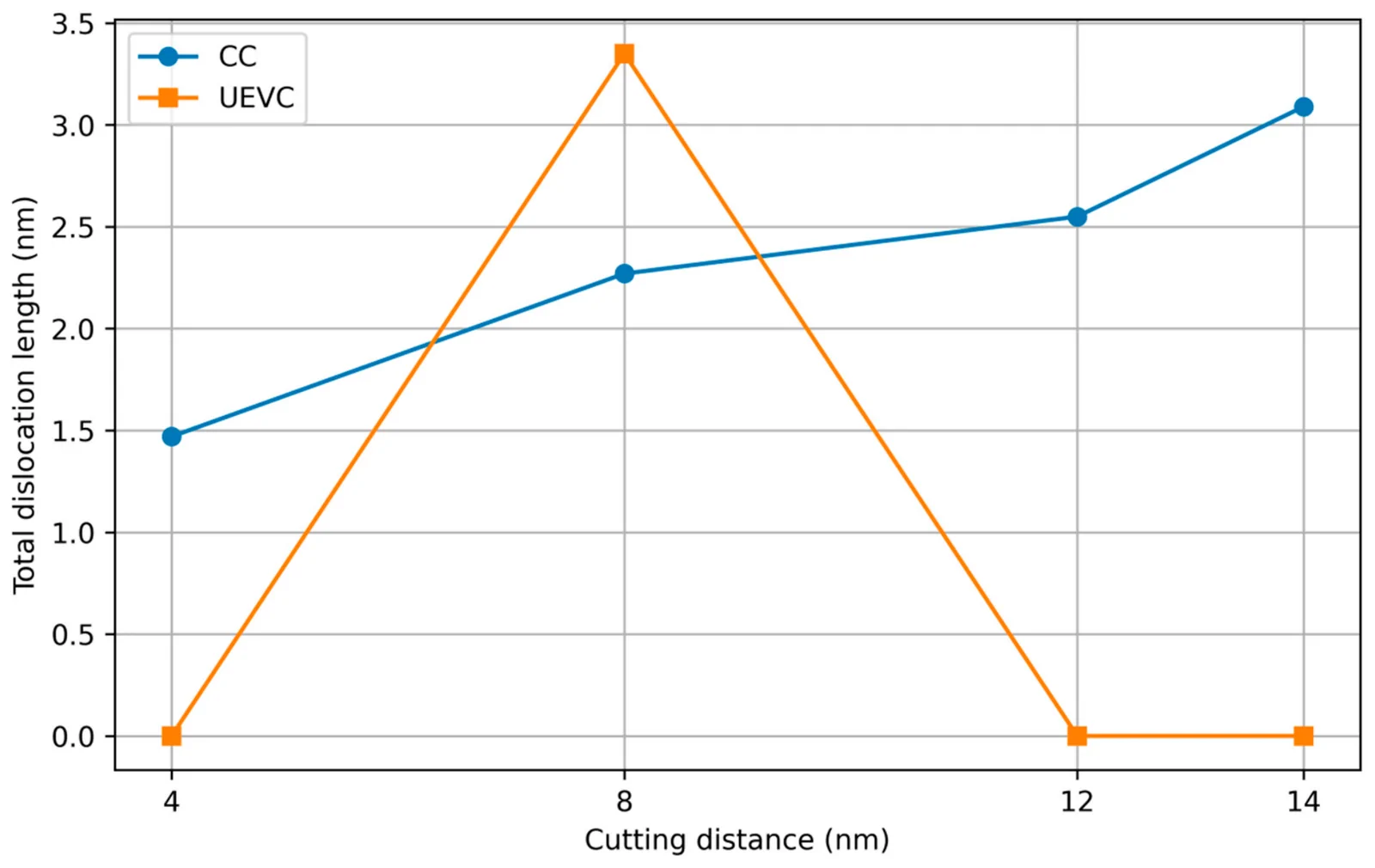
Comparison of dislocation evolution behavior between CC and UEVC at different cutting distances.

As the tool subsequently entered the unloading stage, as shown in Figure 7, no complete DXA-identifiable dislocation lines were detected again at cutting distances of 12 and 14 nm. Accordingly, CC is characterized primarily by the progressive accumulation and retention of dislocations as cutting proceeds, whereas UEVC exhibits transient dislocation activation during specific high-load stages, followed by rearrangement, reconstruction, or loss of well-defined dislocation-line character during the subsequent unloading process. In other words, ultrasonic vibration alters not only the amount of dislocation activity, but also the dynamic evolution pathway of lattice defects in response to time-dependent loading.

It should be emphasized that the disappearance of DXA-identifiable dislocation lines at the later stages of UEVC does not imply the complete elimination of plastic deformation or subsurface structural disorder. A considerable number of atoms in the final atomic configuration remain outside an ideal BCC environment and are mainly distributed within the chip, machined surface, and locally highly distorted regions. Therefore, the results are more appropriately interpreted as a transformation in the form of defect retention under ultrasonic vibration: some defects no longer persist as clear and stable dislocation lines, but instead evolve into localized lattice distortion, structural disorder, and atomic rearrangement. Overall, CC is dominated by progressive dislocation accumulation, whereas UEVC exhibits more pronounced transient dislocation activation and defect reorganization.

As shown in Table 4, to further quantify the non-dislocation local structural evolution, PTM analysis was performed for the UEVC process. The fractions of BCC, HCP, FCC, and other atoms at different cutting distances are summarized in Table X. At 8 nm, the fractions of BCC, HCP, FCC, and other atoms were 39.9%, 0.1%, 0.8%, and 59.2%, respectively. With increasing cutting distance to 12 nm, the BCC fraction increased to 50.2%, while the HCP and FCC fractions remained nearly unchanged (0.2% and 0.8%). At 14 nm, the HCP and FCC fractions further decreased to nearly zero, whereas the other fraction remained approximately 50.9%.

These results indicate that the defect evolution during UEVC is not dominated by a transformation toward HCP/FCC structures. Instead, the structural change is mainly associated with the redistribution between BCC-like configurations and highly distorted local structures, suggesting defect rearrangement and lattice distortion rather than large-scale phase transformation.

### 3.6. Experimental–Atomistic Correlation of Subsurface Deformation

The cross-sectional scanning electron microscopy (SEM) observations obtained in the previously reported experiments provide an important cross-scale reference for the defect analysis performed in the present molecular dynamics simulations. Under conventional cutting conditions, the W–Mo70 alloy exhibited a relatively thick subsurface affected layer, together with typical damage features including a plastically deformed layer, stacking faults, dislocation pile-ups, and transgranular microcracks. Continuous tool–workpiece contact, interfacial friction, and relatively high mechanical loading promoted the progressive accumulation of plastic shear strain and localized deformation within the subsurface region. When the local lattice could no longer accommodate the applied load through further coordinated plastic deformation, the accumulated strain energy was released through mechanisms such as dislocation pile-up, propagation of lattice defects, and eventually crack nucleation and extension, thereby enlarging the subsurface damage zone.

The present molecular dynamics results show good physical correspondence with these experimental observations. Under conventional cutting, the total dislocation length increased continuously from approximately 1.47 nm to 3.09 nm with increasing cutting distance, indicating a pronounced accumulation of lattice defects during continuous cutting. This simulated trend is consistent with the dislocation pile-ups and relatively deep affected layer observed in the experimental cross-sections. While the experiments reveal the final subsurface damage morphology formed after sustained machining at the micrometer scale, the MD simulations further clarify the preceding atomic-scale evolution process, in which dislocations progressively nucleate, propagate, and accumulate before the final damage structure is established. Therefore, the cross-scale analysis indicates that sustained mechanical loading during conventional cutting is a key factor driving the continuous accumulation of subsurface defects.

**Figure 8:**
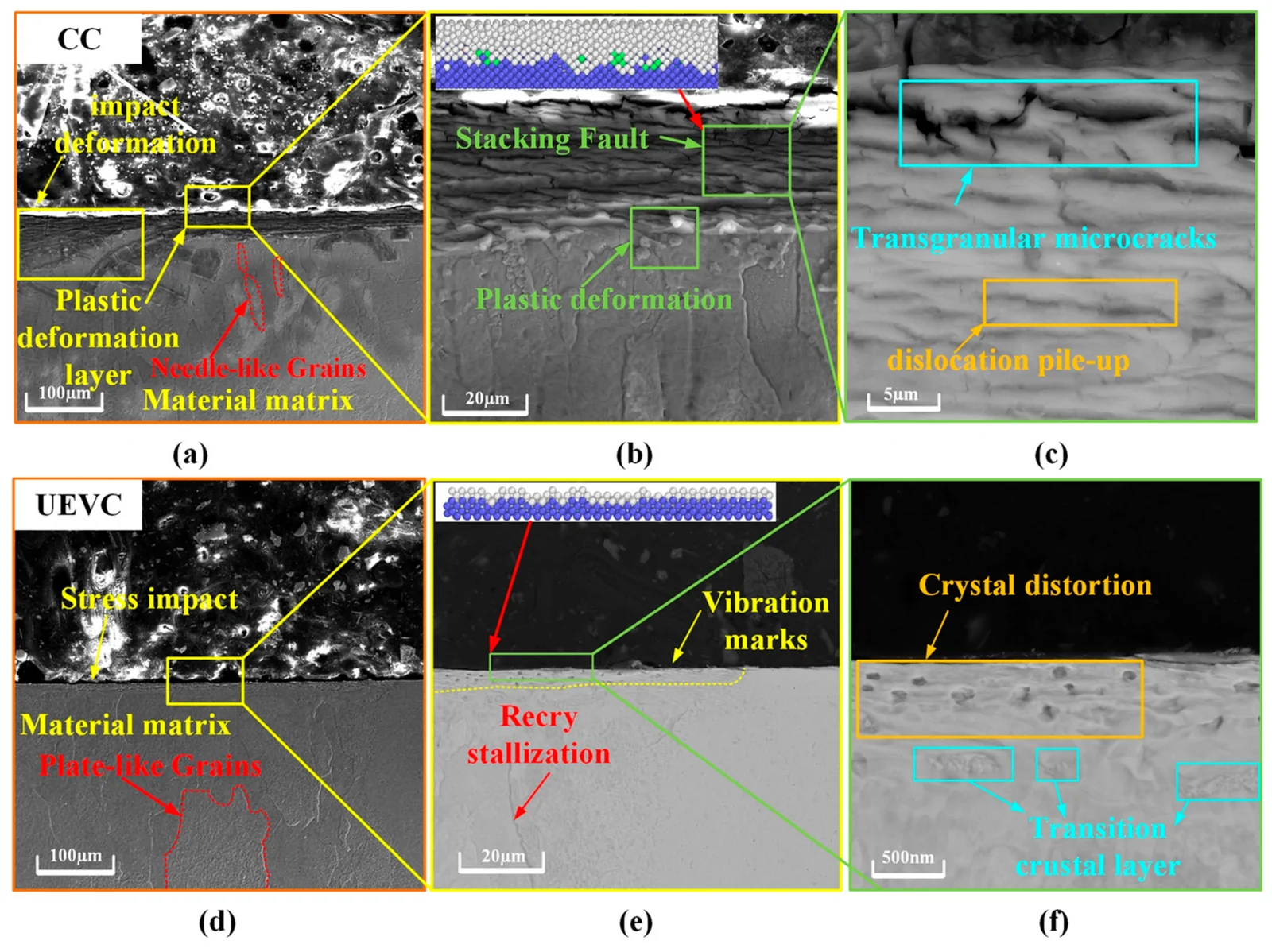
(**a**) Surface and subsurface morphology of CC; (**b**) enlarged view of plastic deformation and stacking faults under CC; (**c**) microstructural defects including transgranular microcracks and dislocation pile-up under CC; (**d**) Surface and subsurface morphology of UEVC; (**e**) enlarged view of recrystallization and vibration-induced marks under UEVC; (**f**) microstructural characteristics including crystal distortion and transition crystal layer under UEVC. Comparison of subsurface SEM microstructures of workpiece after OC ordinary cutting and UEVC ultrasonic vibration-assisted cutting.

Compared with conventional cutting, the subsurface region produced by UEVC exhibited distinct microstructural evolution characteristics in the experiments. As shown in Figure 8, the UEVC-affected subsurface was mainly characterized by vibration-induced traces, lattice distortion, and a transitional crystalline layer, whereas transgranular cracking was no longer the dominant form of subsurface damage. The experimental observations indicate that the cyclic loading and high instantaneous strain rates introduced by ultrasonic vibration promote lattice distortion, atomic rearrangement, and local structural reconstruction near the machined surface. Consequently, part of the plastic deformation can be accommodated through more spatially distributed lattice adjustment mechanisms, thereby limiting the penetration of severe crack-type damage into the workpiece interior.

The dislocation evolution revealed by the MD simulations provides an atomic-scale explanation for these experimental observations. At a nominal cutting distance of 4 nm, no complete DXA-identifiable dislocation lines were detected. At 8 nm, the total dislocation length increased transiently to approximately 3.35 nm, whereas at 12 and 14 nm no complete DXA-identifiable dislocations were observed again. These results indicate that, under UEVC, dislocations do not accumulate monotonically throughout the cutting process. Instead, they are transiently activated during high-load stages and subsequently undergo rearrangement, structural transformation, or loss of a well-defined dislocation line character during the unloading stage. Therefore, the lattice distortion and transitional crystalline layer observed experimentally can be interpreted as the microstructural manifestation of continuous local lattice adjustment and reorganization under periodic loading.

To quantitatively characterize the structural deformation of the machined near-surface region under CC and UEVC conditions, the Polyhedral Template Matching (PTM) method implemented in OVITO was employed to identify the local atomic structures of the W–Mo70 alloy. PTM analysis was performed on the complete atomic configurations prior to geometric cropping to minimize artificial structural misidentification caused by newly generated boundaries. Taking the BCC lattice as the reference crystal structure, W and Mo atoms that were not identified as BCC were collectively classified as non-BCC atoms. Identical PTM parameters and structural classification criteria were applied to both CC and UEVC to ensure direct comparability. Atomic configurations at a nominal cutting distance of 14 nm were selected, and an identical region of interest (ROI) located within the post-machined region was defined as 150 ≤ x ≤ 180 Å and 60 ≤ y ≤ 95 Å. The fraction of non-BCC atoms within the ROI was calculated as follows:

$$f_{\mathrm{non}-\mathrm{BCC}}\;=\;\frac{N_{\mathrm{non}-\mathrm{BCC}}}{N_{\mathrm{ROI}}}\;\times \;100\%$$

where *N*_non−BCC_ denotes the number of non-BCC W/Mo atoms within the ROI, and NROI represents the total number of W/Mo atoms in the corresponding ROI.

**Figure 9:**
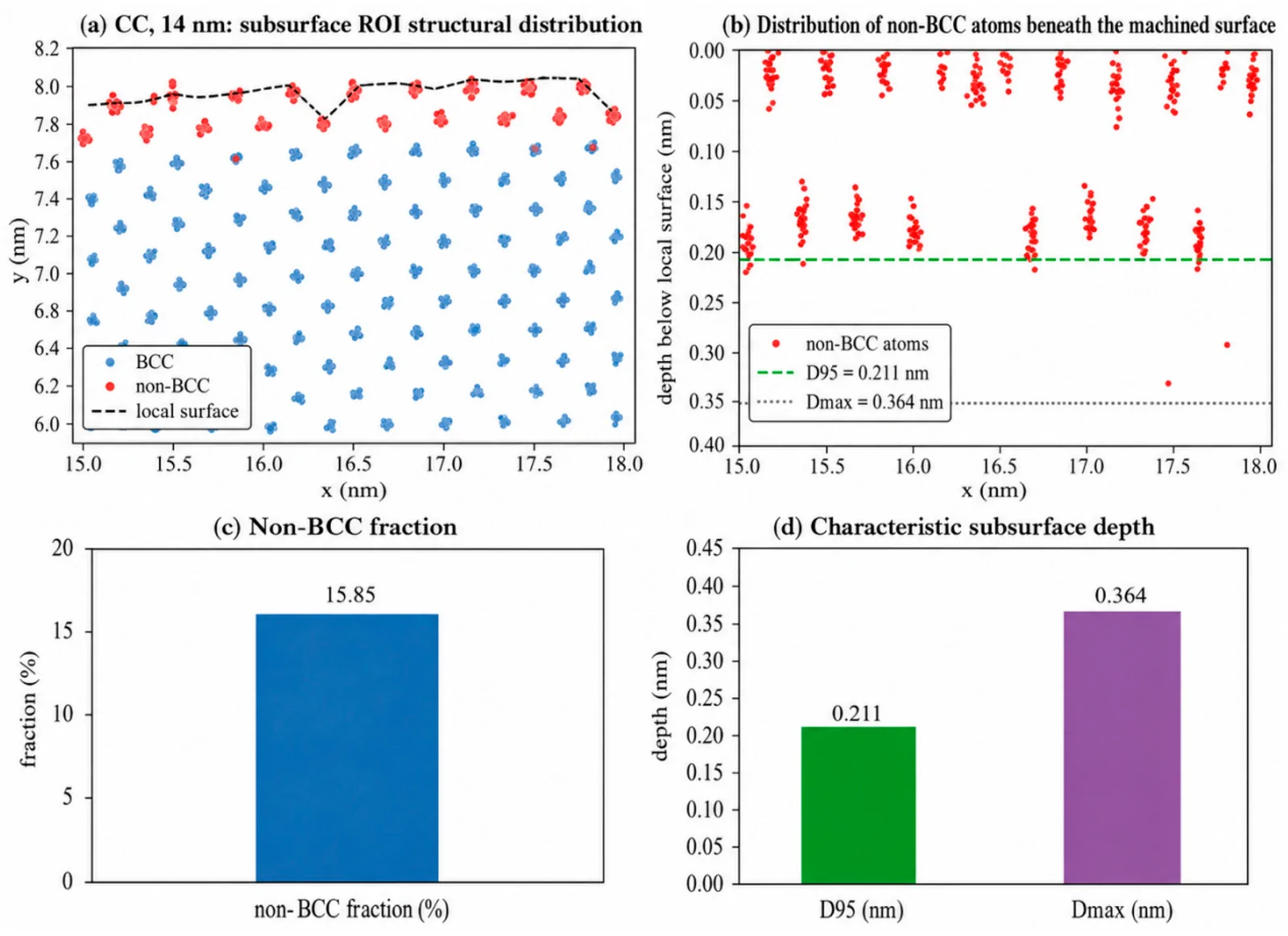
Atomic phase distribution, non-BCC atom fraction and characteristic damage depth in subsurface ROI region for CC at cutting distance of 14 nm.

As shown in Figure 9, the combined experimental and molecular dynamics results reveal distinct subsurface defect evolution modes under the two machining conditions. The dislocation pile-ups and crack-type damage observed experimentally under conventional cutting are physically consistent with the continuously increasing total dislocation length obtained from the MD simulations, both reflecting the progressive accumulation of defects under sustained mechanical loading. In contrast, the structural reorganization characterized by lattice distortion and a transitional crystalline layer under UEVC corresponds well to the transient activation of dislocations and subsequent defect reconstruction revealed by the MD simulations. These observations indicate that the periodic loading–unloading process introduced by ultrasonic vibration fundamentally alters the evolution pathway of lattice defects.

**Figure 10:**
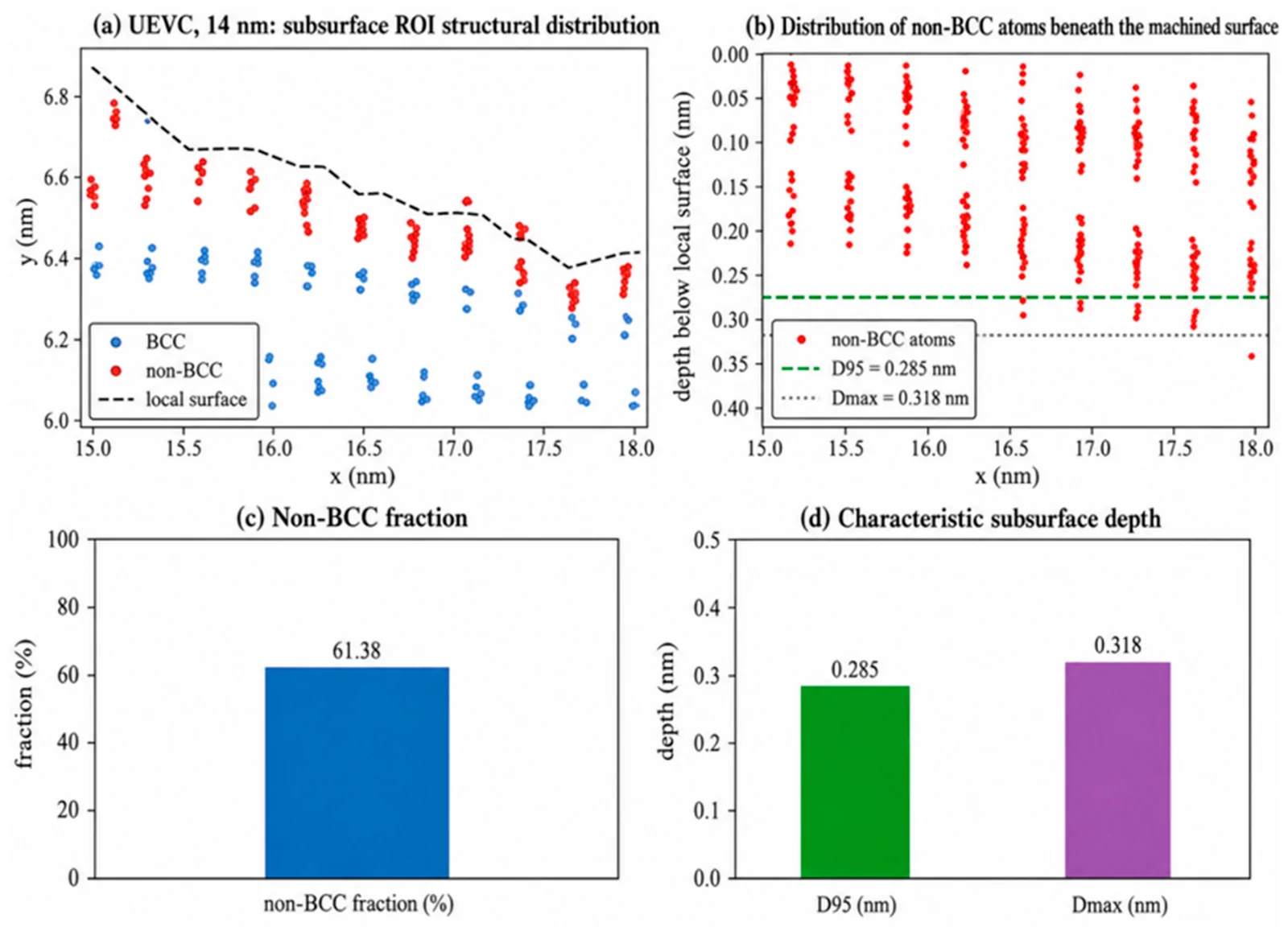
Atomic phase distribution, non-BCC atom fraction and characteristic damage depth in subsurface ROI region for UEVC at cutting distance of 14 nm.

As shown in Figure 10, the beneficial effect of ultrasonic vibration on subsurface deformation should not be interpreted simply as a reduction in the number of defects at all stages of the cutting process. Instead, ultrasonic vibration periodically modulates the tool–workpiece interaction, thereby altering the mechanisms governing defect generation, accumulation, and reorganization. Compared with the progressive accumulation of defects under conventional cutting, UEVC tends to accommodate localized deformation through transient dislocation activity, lattice rearrangement, and structural reorganization. Consequently, the periodic loading–unloading behavior modifies both the formation mechanism and spatial distribution of subsurface damage.

### 3.7. Mechanism of Ultrasonic-Assisted Ultra-Precision Cutting

By integrating the experimental observations and molecular dynamics simulation results, the deformation and defect evolution mechanisms of the W-Mo70 alloy under CC and UEVC are systematically summarized in Figure 9.

**Figure 11:**
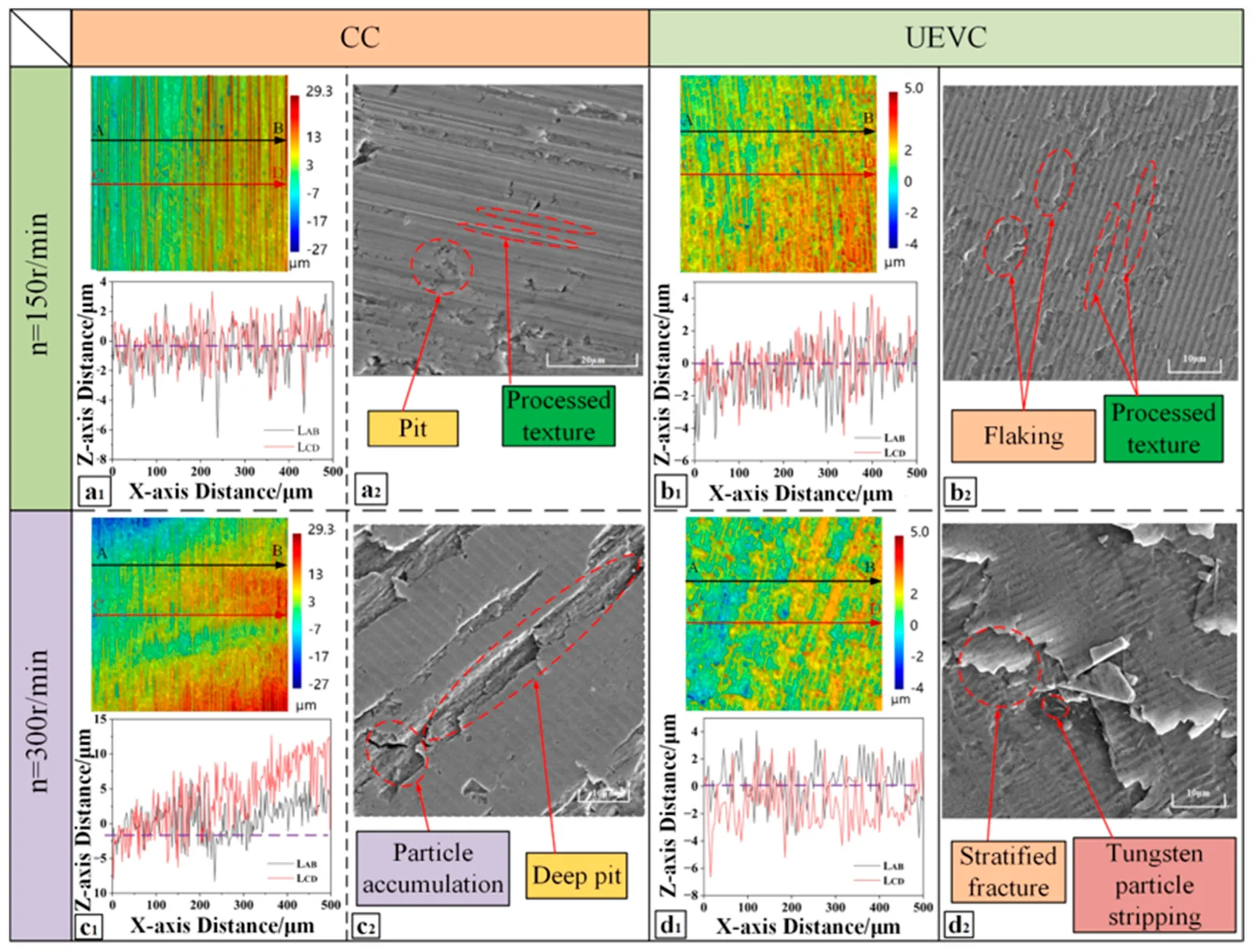
(**a_1_**) Three-dimensional surface morphology and roughness profile of CC at n = 150 r/min; (**a_2_**) SEM image showing pits and processed texture under CC at n = 150 r/min; (**b_1_**) Three-dimensional surface morphology and roughness profile of UEVC at n = 150 r/min; (**b_2_**) SEM image showing flaking and processed texture under UEVC at n = 150 r/min; (**c_1_**) Three-dimensional surface morphology and roughness profile of CC at n = 300 r/min; (**c_2_**) SEM image showing particle accumulation and deep pits under CC at n = 300 r/min; (**d_1_**) Three-dimensional surface morphology and roughness profile of UEVC at n = 300 r/min; (**d_2_**) SEM image showing stratified fracture and tungsten particle stripping under UEVC at n = 300 r/min. White-light interference topography, profile curves and SEM micro-characteristics of machined surfaces under OC ordinary cutting and UEVC ultrasonic vibration-assisted cutting at different rotational speeds.

As shown in Figure 11, pronounced differences can be observed in the surface topographies, profile curves, and SEM morphologies obtained under CC and UEVC conditions. Under conventional cutting, continuous tool–workpiece interaction promotes the accumulation of machining marks, localized pits, and surface damage due to sustained mechanical loading. In contrast, UEVC periodically changes the contact state between the tool and workpiece, resulting in a more localized and non-steady-state material removal process. Although transient impact during ultrasonic vibration may still induce localized material accumulation or detachment, the overall surface formation behavior is significantly modified compared with continuous cutting.

During conventional cutting, the workpiece material is continuously subjected to compressive, shear, and interfacial frictional loading. With increasing cutting distance, local stress and lattice strain progressively accumulate near the cutting region, promoting continuous dislocation nucleation, extension, and retention. The MD results show that the total length of DXA-identifiable dislocations increases with cutting distance, indicating progressive defect accumulation under sustained mechanical interaction. This behavior is consistent with the experimentally observed plastically deformed layer, dislocation pile-ups, and transgranular microcracks, where accumulated plastic strain is accommodated through persistent defect generation and propagation.

In contrast, UEVC periodically modulates the instantaneous penetration state of the tool relative to the workpiece. During the loading stage, enhanced tool penetration generates high transient stresses, promoting localized lattice shear and dislocation activation. When the tool enters the unloading stage, the reduced tool–workpiece interaction provides opportunities for stress relaxation, atomic rearrangement, and defect restructuring. Therefore, unlike the continuous accumulation of defects under CC, UEVC induces a transient defect evolution process characterized by activation during high-load stages and subsequent rearrangement during unloading. The DXA and PTM analyses indicate that the disappearance of well-defined DXA dislocation lines at later stages is associated with defect restructuring and local lattice distortion rather than large-scale HCP/FCC structural transformation.

The combined experimental and simulation results suggest that the fundamental mechanism of UEVC is governed by periodic loading, interfacial unloading, defect reorganization, and localized material removal. Ultrasonic vibration does not simply eliminate plastic deformation or reduce all instantaneous loads; instead, it changes the pathway of deformation by transforming sustained stress accumulation into a cyclic process involving defect activation, stress relaxation, and structural adjustment. This modification of the deformation pathway provides an atomic-scale explanation for the reduction in average cutting resistance and the improved machinability of the W-Mo70 alloy.

It should be noted that differences exist between the experimental material and the present MD model. The experimental W-Mo70 workpiece is a polycrystalline rod containing grain boundaries and pre-existing defects, whereas the MD model represents an idealized BCC random solid solution without these microstructural features. Therefore, experimentally observed grain boundary-related phenomena, such as dislocation pile-ups and crack initiation, cannot be directly reproduced by the current MD model. In addition, the calculated kinetic temperature represents atomic kinetic activity rather than a rigorous thermodynamic temperature because the streaming velocity contribution is not explicitly removed. Therefore, the experimental comparison is mainly used to validate the overall cutting-response trend, while the MD simulations provide atomic-scale insights into the deformation and defect evolution mechanisms of UEVC.

**Figure 12:**
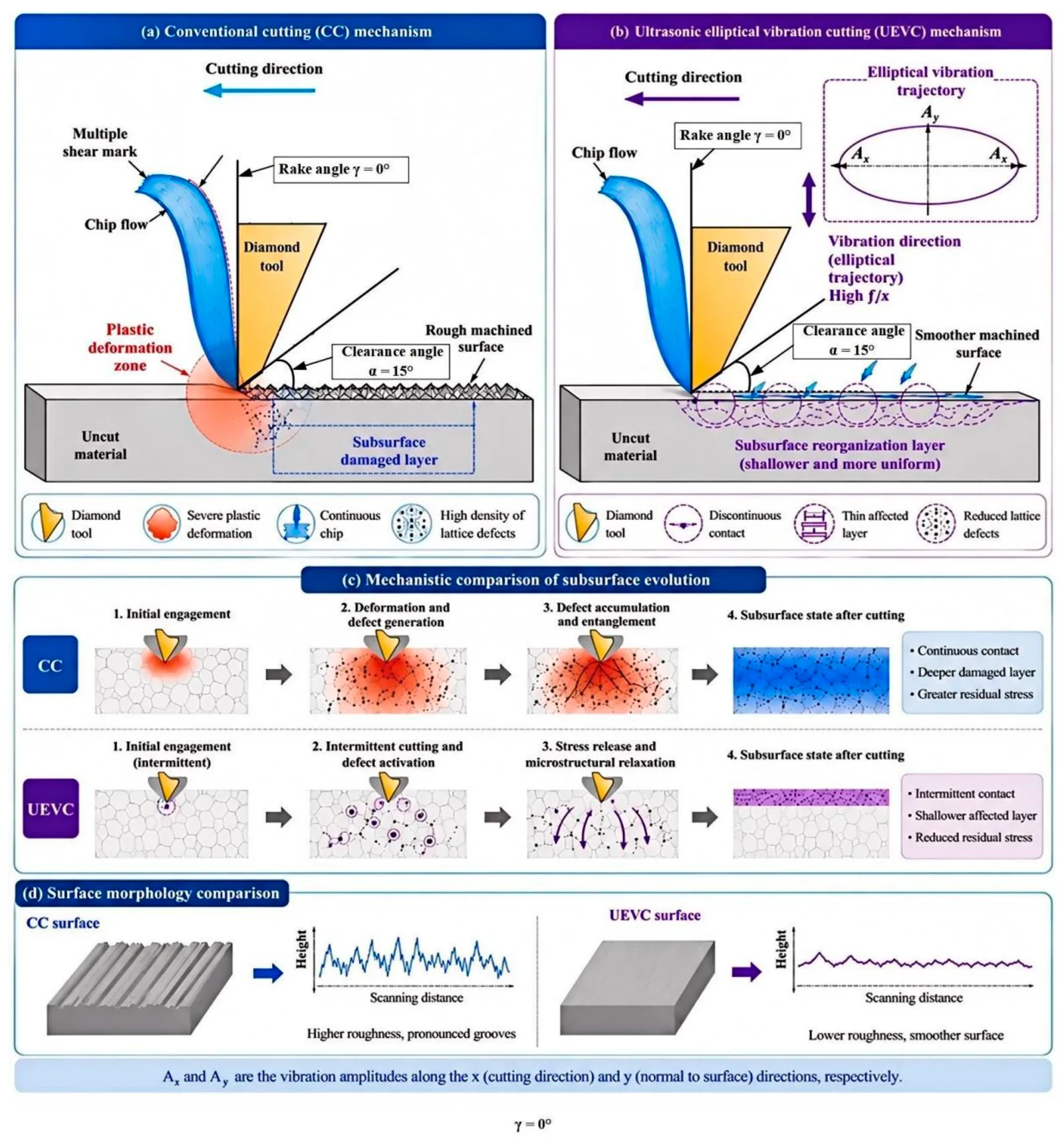
Schematic diagram comparing machining mechanism, subsurface evolution and surface morphology between CC and UEVC.

As illustrated in Figure 12a, under conventional cutting, the tool maintains continuous contact with the workpiece, resulting in sustained compressive, shear, and frictional loading at the tool–workpiece interface. As cutting proceeds, continuous mechanical interaction causes progressive accumulation of local stress and lattice strain in the subsurface region. This promotes continuous dislocation nucleation, propagation, and retention, leading to the development of a highly damaged subsurface layer.

In contrast, as shown in Figure 12b, UEVC introduces an elliptical vibration trajectory superimposed on the primary cutting motion, periodically changing the instantaneous penetration state and contact condition between the tool and workpiece. During the loading stage, the tool penetrates into the material and induces transient high stresses, activating localized plastic deformation and defect generation. During the subsequent unloading stage, reduced tool–workpiece interaction provides opportunities for stress relaxation, atomic rearrangement, and defect restructuring.

The different subsurface evolution pathways under CC and UEVC are further summarized in Figure 12c. Under CC, continuous loading results in progressive defect accumulation and subsurface damage development. In comparison, UEVC transforms the deformation process into a cyclic pathway involving intermittent loading, defect activation, stress relaxation, and structural adjustment.

As shown in Figure 12d, the continuous contact condition during CC produces relatively continuous machining marks and higher surface damage accumulation, whereas the intermittent contact characteristics of UEVC modify the material removal behavior and result in a more localized and non-steady-state surface formation process.

It should be noted that differences exist between the experimental material and the present MD model. The experimental W-Mo70 workpiece is a polycrystalline rod with grain boundaries and pre-existing defects, whereas the MD model is an idealized BCC random solid solution without these microstructural features. Therefore, grain boundary-related deformation behaviors, such as dislocation pile-ups and transgranular crack initiation, cannot be directly reproduced. The experimental comparison is therefore used to validate the overall cutting-response trends, while the MD simulations mainly provide atomic-scale insights into the deformation and defect evolution mechanisms of UEVC. Therefore, the identified defect evolution pathway should be interpreted as a representative atomic-scale response within the captured vibration cycle, while further simulations involving multiple vibration cycles are required to evaluate long-term statistical reproducibility.

## 4. Conclusions

1. The experimental results and molecular dynamics simulations exhibit good agreement in the overall trend of cutting force variation. The previously reported ultra-precision turning experiments showed that the major cutting force components were significantly reduced after the introduction of UEVC. Consistently, the MD simulations demonstrate that UEVC decreases the cycle-averaged main cutting force compared with CC. Although the exact reduction magnitude may vary due to atomic-scale randomness, the consistent reduction trend confirms the effect of ultrasonic vibration on reducing cutting resistance, cutting speed, vibration frequency, and tool dimensions, both demonstrate that ultrasonic vibration reduces the average main cutting resistance, while the MD model is mainly used to elucidate the corresponding atomic-scale mechanisms rather than reproduce the experimental cutting force magnitude.

2. The primary effect of UEVC on the tool–workpiece interface is vibration-induced transient loading–unloading response redistribution rather than a continuous reduction in cutting forces in all directions. Under CC, the normal load progressively develops as cutting proceeds, whereas under UEVC, the normal force exhibits a characteristic increase–peak–decrease–reloading pattern with vibration phase. Its cycle-averaged value is approximately 774 nN, which is nearly identical to the 771 nN obtained under CC, while the instantaneous normal force can decrease rapidly from about 1600 nN to approximately 69 nN. Meanwhile, UEVC produces a higher instantaneous atomic kinetic response, indicating that periodic vibration significantly enhances the non-steady-state and phase-dependent characteristics of the cutting process.

3. Ultrasonic vibration significantly alters the evolution of subsurface dislocations in W-Mo70. Under CC, the DXA-identifiable dislocation length increases from approximately 1.47 to 3.09 nm with cutting distance, indicating progressive defect accumulation. In contrast, UEVC induces transient dislocation activation, followed by defect rearrangement during unloading, whereas no complete DXA-identifiable dislocation lines are detected at the subsequent 12 and 14 nm stages. This observation does not necessarily indicate the complete elimination of defects, because highly distorted atomic configurations or locally disordered regions may reduce the reliability of BCC-based DXA identification. Although PTM analysis was not performed in the present study, the calculated kinetic temperature remains below the melting point of W-Mo70 alloy. Therefore, the disappearance of DXA-identifiable dislocation lines is interpreted cautiously as a loss of detectable stable dislocation structures rather than complete defect elimination. These results indicate that UEVC does not continuously suppress dislocation formation; instead, it transforms defect evolution from progressive accumulation into transient activation during high-load stages followed by rearrangement and structural reorganization during unloading.

4. By integrating the experimental observations with the MD results, the improved machinability of W-Mo70 under UEVC is attributed to the modified tool–workpiece interaction and altered subsurface defect evolution. The experimental and simulation results consistently demonstrate that ultrasonic vibration reduces average cutting resistance and changes the defect evolution pathway from persistent accumulation toward transient activation and structural adjustment. In contrast, UEVC periodically modulates the tool penetration state, promoting localized plastic activation during high-load stages while providing opportunities for atomic rearrangement and defect structure adjustment during subsequent unloading. This changes the pathway of persistent subsurface defect accumulation and provides an atomic-scale explanation for the reduction in average cutting resistance and the improved machinability of W-Mo70.

## Figures and Tables

**Table 1:** Chemical composition and physical properties of the investigated materials.

Material	W Content	Mo Content	Workpiece Dimension	Density	Melting Point	Thermal Conductivity
W-Mo	30 at.%	70 at.%	Φ25 × 75 mm	16,500 kg/m^3^	2800 °C	125 W/(m·K)

**Table 2:** Geometrical parameters of tool.

Shape	Side Length/mm	Thickness/mm	Accuracy	Nose Radius/mm	Rake Angle/°	Relief Angle/°
Triangle	9.6	2.38	G	1.2	0	15

**Table 3:** Key simulation parameters of molecular dynamics cutting models.

Category	Parameter	Value/Description
Workpiece	Composition	W-Mo70 (30 at.% W, 70 at.% Mo), BCC random substitutional solid solution
	Dimensions	15.00 × 9.96 × 10.13 nm (x × y × z)
	Boundary conditions	x/y: non-periodic; z: periodic
	Temperature & ensemble	Initial 300 K; NVT relaxation, Langevin thermostat removed during cutting stage
	Region partition	Fixed boundary layer, thermostatic layer, deformable mobile region
Tool	Material & constraint	Diamond, rigid body
	Edge radius	~1.43 nm
Interatomic potentials	W–W/Mo–Mo/W–Mo	EAM/FS potential [31]
	C–C (diamond)	Tersoff potential [32]
	C–W/C–Mo interface	Lennard–Jones potential, ε = 0.04 eV, σ = 3.0 Å, cutoff distance = 10 Å
Motion parameters	CC speed	250 m/s
	UEVC vibration amplitude	Ax = Ay = 1.5 nm
	UEVC vibration frequency	20 GHz (vibration period: 50 ps)
	Amplitude ratio λ	1:1
Simulation settings	Integration time step	1 fs
	Thermal relaxation time	20 ps
	Total cutting duration	57.5 ps (~1.15 complete ultrasonic vibration cycles)

**Table 4:** Evolution of local lattice structures identified by PTM during UEVC at different cutting distances.

Cutting Distance	BCC (%)	HCP (%)	FCC (%)
8 nm	39.9	0.1	0.8
12 nm	50.2	0.2	0.8
14 nm	49.0	0.0	0.1

## Data Availability

The data presented in this study are available on request from the corresponding author.
